# Inequalities in the geographic distribution of hospital beds and doctors in traditional Chinese medicine from 2004 to 2014

**DOI:** 10.1186/s12939-018-0882-1

**Published:** 2018-11-12

**Authors:** Liming Lu, Jingchun Zeng

**Affiliations:** 1 Clinical Research Center, South China Research Center for Acupuncture and Moxibustion, Medical College of Acu-Moxi and Rehabilitation, Guangzhou University of Chinese Medicine, Guangzhou, 510006 China; 2 Department of Acupuncture, the First Affiliated Hospital of Guangzhou University of Chinese Medicine, Guangzhou, 510405 China

**Keywords:** China, Health care resources, Traditional Chinese medicine (TCM), Inequality

## Abstract

**Objectives:**

This study identifies inequities in the provincial-level geographical distribution of traditional Chinese Medicine (TCM) hospital beds and doctors in China from 2004 to 2014. This provides policy implications of the optimal allocation of TCM health care resources.

**Methods:**

Our study used province level data on TCM hospital beds and doctors from 2004 to 2014. These data were obtained from the China TCM Yearbook 2004–2014 and the China Statistical Yearbook 2004–2014.Global and local spatial autocorrelation was performed by using Moran’s index and the local Moran’s index to describe the spatial distribution of TCM hospital beds (doctors) as well as their density. A Gini coefficient was used to estimate inequalities in the geographic distribution of TCM hospital beds (doctors) based on their density. Correlations of the Gini coefficients between TCM hospital beds and doctors were calculated by Pearson correlation analysis.

**Results:**

All indicators of TCM hospital beds and doctor density have increased over the past 11 years. The number of TCM hospital beds per 10,000 populations increased the fastest. Geographical clustering was not obvious in the density distribution of TCM hospital beds or doctors, as no significant spatial autocorrelation was found. Gini coefficients showed that from 2004 to 2014 the distribution of TCM hospital beds per 10,000 population and doctors per 10,000 populations were equitable between different regions. A large gap existed in the distribution inequality of TCM hospital beds (doctors) per square kilometer among different regions.

**Conclusion:**

Targeted health policy with equitable distribution of TCM hospital beds (doctors) per square kilometer and the balance and coordination of related resources should be a priority in shaping China’s healthcare system reform.

## Introduction

At present, healthcare reform in many countries aims to provide universal and equitable health care access, and health equity is believed to be an important goal of healthcare reform [1–3]. However, inequalities in the geographic distribution of healthcare resources have limited the sustainable development of health system quality, health outcomes and economic efficiency [4–6].

Demand for traditional Chinese Medicine (TCM) services in China is immense. The total number of people (including inpatients and outpatients) in China who visit TCM hospitals has increased every year, from 170 million in 2009to 471 million in 2014; the number of inpatients discharged from TCM hospitals has also increased every year, from 9.75 million in 2009 to 20 million in 2014 [7]. In February 2016, China’s State Council issued an outline of strategic planning for the development of traditional Chinese Medicine (TCM) [8]. In this plan, the Chinese government pointed out that the demand for TCM services still exceeds supply due to limited TCM healthcare resources and the availability of services. The goal of universal access to TCM services by the end of 2020 was proposed (the number of beds per 1000 population in public TCM hospitals should reach 0.55, and doctors per 1000 population should reach 0.4) and it is an urgent task to reasonably allocate the distribution of human care resources and effectively improve the equitable distribution of these resources [8]. In China, due to the unbalanced development of urban and rural areas, health personnel have tended to start their careers in economically developed rather than poor areas. Therefore a large number of TCM hospital beds have been allocated to cities [9]. Among indicators of basic public health services, Gini coefficients for TCM were the largest in 2014, meaning that serious inequalities existed in the distribution of TCM [10]. Scant evidence exists to reveal the inequality in the geographic distribution of TCM hospital beds and doctors that has persisted for many years [11, 12].

Our study aimed to 1) depict inequities in the geographical distribution of TCM hospital beds and doctors in China from 2004 to 2014; 2) provide policy implications for the optimal allocation of TCM hospital beds and doctors to improve the development of TCM services for the Chinese government.

## Methods

### Data sources

This study used province-level data on TCM hospital doctors and beds from 2004 to 2014. These data were obtained from the China TCM Yearbook 2004–2014 (number of resident population and size of provinces) and the China Statistical Yearbook 2004–2014 (number of TCM Hospital beds (doctors) of provinces), respectively published by the Department of State Administration of Traditional Chinese Medicine and the National Bureau of Statistics of China. These yearbooks report province level data annually from 31 Chinese provinces, and do not include Hong Kong Special Administrative Region (SAR), Macao SAR or Taiwan.

### Variable(s) of interest

Previous reports have used the density of TCM hospital beds and doctors as proxy metrics for healthcare resources [13]. The ratio of TCM Hospital beds (doctors) per 10,000 population was calculated based on the total number of beds (doctors) in TCM hospitals and health centers per 10,000 resident population in that province at the end of the year [12]. Similarly, TCM hospital beds (doctors) per square kilometer was calculated based on the total number of beds (doctors) in TCM hospitals and health centers divided by land area of each province at the end of the year. According to the statistical standards of the Yearbook, regular beds and nursing beds were included, however, pre-delivery beds, beds in outpatient observation rooms and in obstetric wards for newborn babies were not. TCM doctors included professionals who practiced TCM and held both a TCM physician qualification certificate and a physician practicing certificate in China.

### Ethics statement

The data from the Statistical Yearbook are publicly available. Informed consent was not needed, as these were secondary data without any personal information.

### Data analysis

#### Global and local spatial autocorrelation analyses

To assess the correlation between TCM hospital beds (doctors) density and their spatial location, Moran’s index (Moran’s *I*) and local Moran’s index (local Moran’s *I*_*i*_) were calculated for global and local spatial autocorrelation, respectively [14–18]. We used Global Moran’s *I* to evaluate the entire degree of spatial autocorrelation, and the local Moran’s *I*to estimate the local autocorrelation between a single position and its neighbors.

The equation for calculating Global Moran’s *I* was as follows:

1 $$ I=\frac{n}{\sum_{i=1}^n{\sum}_{j=1}^n{w}_{ij}}\frac{\sum_{i=1}^{\mathrm{n}}{\sum}_{j=1}^n{w}_{ij}\left({x}_i-\overline{x}\right)\left({x}_j-\overline{x}\right)}{\sum_{i=1}^n{\left({x}_i-\overline{x}\right)}^2}\kern0.75em i\ne j $$

where *n* is the total number of provinces (*n* = 31), *x*_*i*_ and *x*_*j*_ were TCM hospital beds (doctors) density of the *i*th and *j*th province of interest, $\overline{x}$ is the average TCM hospital beds (doctors) density of 31 provinces, and *w*_*ij*_ refers to the spatial weight between locations *i* and *j*.

Similarly, the equation for calculating Local Moran’s *I* was as follows:

2 $$ I=\frac{n\left({x}_i-\overline{x}\right){\sum}_{j=1}^n{w}_{ij}\left({x}_j-\overline{x}\right)}{\sum_{i=1}^n{\left({x}_i-\overline{x}\right)}^2}\kern0.75em i\ne j $$

The explanation of parameters in Eq. (2) was the same as those in Eq. (1). The map of local spatial autocorrelation can be classified as follows (*a* = 0.05): high-value cluster with high TCM hospital beds (doctors) density (HH), low-value cluster with low TCM hospital beds (doctors) density (LL), low-value outlier with high TCM hospital beds (doctors) density (HL), or high-value outlier with low TCM (LH).The software GeoDa (https://geodacenter.asu.edu/software) was used for spatial analysis.

#### Gini coefficient evaluation

A Gini coefficient was used to estimate inequality in the geographic distribution of TCM hospital beds (doctors) density. Gini coefficients range from 0 to 1: the higher the value, the greater the inequality [19–26]. Different Gini values indicate different equality: ≤0.2 absolute equality, > 0.2 relative equality, 0 > 0.3 reasonably equal; > 0.4 a bigger difference, > 0.5 a large gap [11, 27].

#### Correlations of Gini coefficients between TCM hospital beds and doctors

Pearson correlation analysis was used to explore the correlation between the Gini coefficients of TCM hospital beds and doctors**.** This analysis aimed to test whether the government made a health policy decision considering the allocation of health workforce and beds together, as the management of TCM hospital beds requires a corresponding health workforce.

Gini coefficients and their correlations were calculated by Stata statistical software version 12.0 (StataCorp LP, College Station, TX, U.S.). Inequality figures based on Gini coefficients and their correlations were performed by R software, version 3.3 (R Project for Statistical Computing).

## Results

### Distribution trends for TCM hospital beds and doctors from 2004 to 2014

**Fig. 1:**
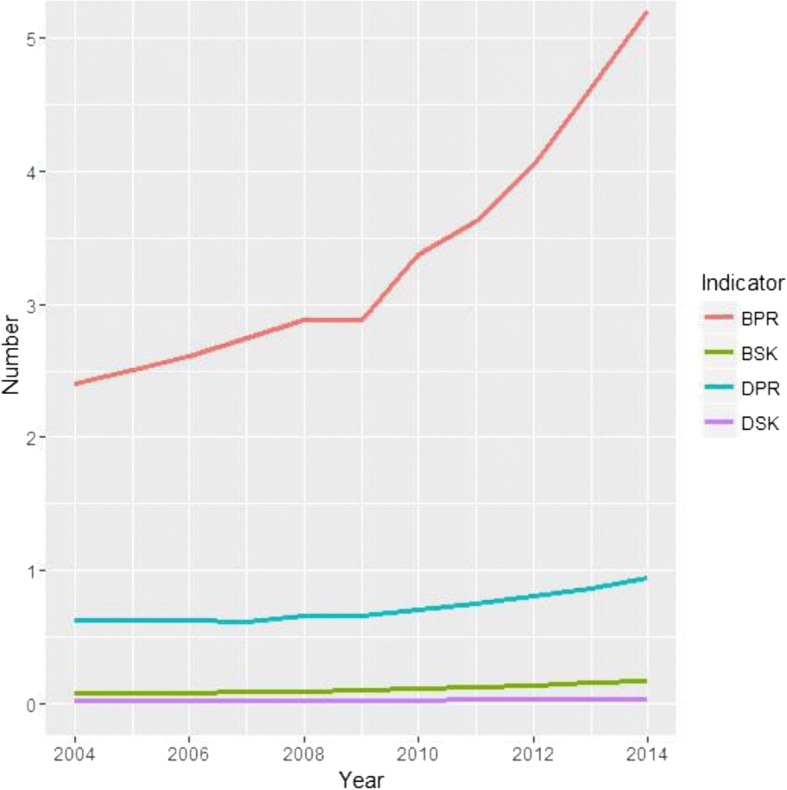
Distribution trends for TCM hospital beds and doctors from 2004 to 2014. BPP, TCM hospital beds per 10,000 population; BSK, TCM hospital beds per square kilometer; DPP, TCM doctors per 10,000 population; DSK, TCM doctors per square kilometer

On average over the 11 year period, there were was a ratio of 3.35 TCM hospital beds per 10,000 population, 0.72 doctors per 10,000 population, 0.11 TCM hospital beds per square kilometer and 0.03 doctors per square kilometer. From 2004 to 2014, the ratios of TCM hospital beds per 10,000 population, doctors per 10,000 population, TCM hospital beds per square kilometer and doctors per square kilometer all increased from 2.41 to 5.21, 0.63 to 0.94, 0.08 to 0.18 and 0.02 to 0.04, respectively. Of these, the ratio of TCM hospital beds per 10,000 population rose the fastest (Fig. 1).

### Distribution of TCM hospital beds and doctors at the provincial level

**Fig. 2:**
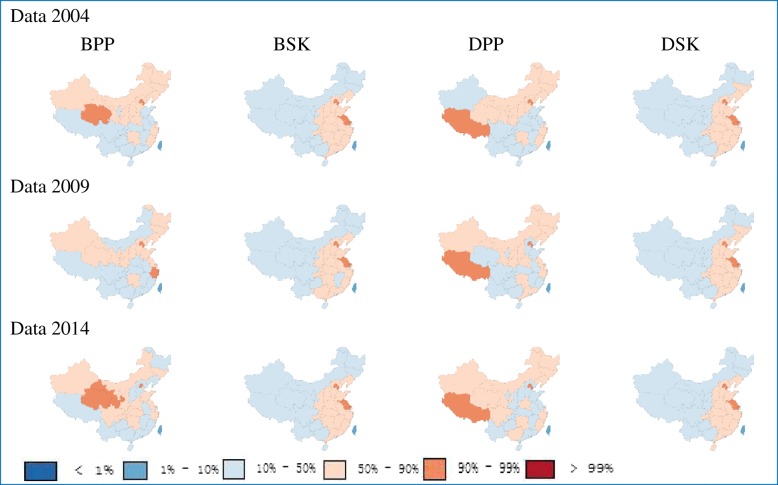
Distribution of TCM hospital beds and doctors at the provincial level. BPP, TCM hospital beds per 10,000 population; BSK, TCM hospital beds per square kilometer; DPP, TCM doctors per 10,000 population; DSK, TCM doctors per square kilometer

Figure 2 contrasts two different measures of hospital beds and doctor density. For TCM hospital beds per 10,000 population, Western counties with darker red had the highest bed densities. TCM hospital beds per square kilometer, known as spatial distance to service provider, were split by unit area to give another picture of a determinant of health demand. Based on this indicator, the reversed trend was observed that higher bed density existed in Eastern provinces while Western areas had lower bed density. Similarly, we also observed this trend for doctors per 10,000 population and doctors per square kilometer. The distribution of indicators mentioned above showed the same trend from 2004 to 2014. For space constraints, only data from 2004, 2009 and 2014 are presented in Fig. 2.

### Global and local spatial autocorrelation analyses

In Table 1, we did not find any significant spatial autocorrelation in TCM hospital beds per 10,000 population, TCM hospital beds per square kilometer, TCM doctors per 10,000 population or TCM doctors per square kilometer from 2004 to 2014 (all *P* > 0.05). The lone exception was TCM hospital beds per 10,000 population in 2011 (*P* < 0.05).

**Table 1:** Global spatial autocorrelation analysis of TCM hospital beds and doctors distribution from 2004 to 2014

Year	TCM Hospitals beds per 10,000 population			TCM Doctors per 10,000 population			TCM Hospital beds per square kilometer			TCM Doctors per square kilometer		
	Moran’s I	Z	*P*	Moran’s I	Z	*P*	Moran’s I	Z	*P*	Moran’s I	Z	*P*
2004	0.11	1.77	0.09	0.04	0.64	0.53	−0.01	− 0.21	0.83	− 0.03	− 0.58	0.57
2005	0.08	1.28	0.21	0.05	0.70	0.49	−0.01	− 0.22	0.83	− 0.03	− 0.61	0.54
2006	0.08	1.34	0.19	0.05	0.74	0.47	−0.01	− 0.23	0.82	− 0.03	−0.64	0.53
2007	0.07	1.23	0.23	0.05	0.76	0.45	−0.02	−0.29	0.78	−0.03	−0.55	0.58
2008	0.08	1.43	0.16	0.06	0.83	0.41	−0.01	−0.22	0.83	−0.03	−0.61	0.54
2009	0.08	1.43	0.16	0.05	0.68	0.50	0.00	0.02	0.99	−0.03	−0.62	0.54
2010	0.10	1.74	0.09	0.05	0.78	0.44	−0.01	−0.14	0.89	−0.03	−0.60	0.55
2011	0.13	2.37	0.02^*^	0.04	0.58	0.56	0.00	0.03	0.98	−0.03	−0.66	0.52
2012	0.07	1.33	0.19	0.04	0.62	0.54	0.01	0.18	0.86	−0.03	−0.66	0.51
2013	0.05	1.05	0.30	0.03	0.53	0.60	0.01	0.16	0.88	−0.03	−0.67	0.51
2014	0.07	1.46	0.15	0.04	0.64	0.53	0.01	0.25	0.81	−0.03	−0.60	0.55

“Z” is a statistic that is approximately normally distributed under the null hypothesis

^*^P ≤ 0.05

**Fig. 3:**
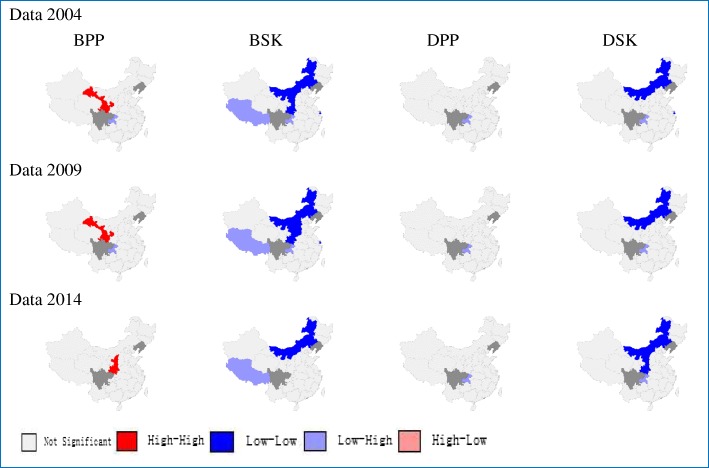
Clusters of Local Moran’s I analysis from 2004 to 2014. BPP, TCM hospital beds per 10,000 population; BSK, TCM hospital beds per square kilometer; DPP, TCM doctors per 10,000 population; DSK, TCM doctors per square kilometer

Local spatial analysis observed a statistically significant clustering of districts into “hotspots” and “coldspots” of indictors (Fig. 3).The Local Moran’s I showed that the core clustering of districts with high TCM hospital beds per 10,000 population next to high ones (HH) from 2004 to 2014were consistently located in Gansu and Shaanxi. Analysis also showed a core “coldspot” cluster of low-next-to-low (LL) districts consistently located in Inner Mongolia and Shaanxi from for TCM hospital beds per square kilometer and doctors per square kilometer from 2004 to 2014.

### Inequality trends based on the Gini coefficient method in different regions

**Fig. 4:**
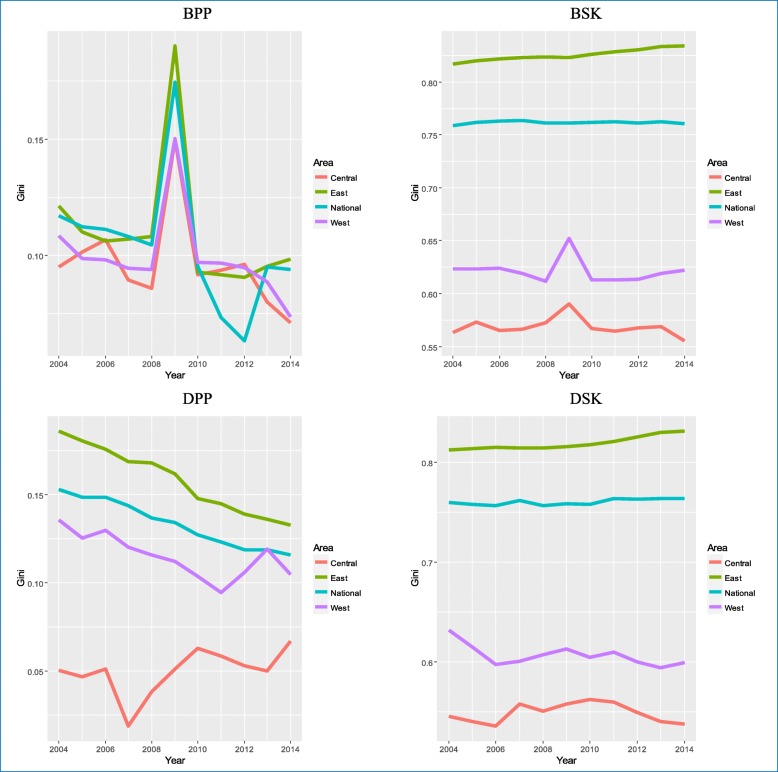
Inequality trends based on the Gini coefficient method in different regions. BPP, TCM hospital beds per 10,000 population; BSK, TCM hospital beds per square kilometer; DPP, TCM doctors per 10,000 population; DSK, TCM doctors per square kilometer

Figure 4 shows that though the Gini coefficients from 2004 to 2014 presented different changes for different regions (East, West, Central and National), all Gini coefficients were less than 0.2 for TCM hospital beds per 10,000 population and doctors per 10,000 population. However, all Gini coefficients were higher than 0.5 from 2004 to 2014 among different regions for TCM hospital beds per square kilometer and doctors per square kilometer. Of note, all Gini coefficients were above 0.8 during these 11 years in the East; above 0.7 nationally; above 0.55 in the West; and above 0.5 in the Central.

### Correlations between Gini coefficients of TCM hospital beds and doctors in different regions

**Fig. 5:**
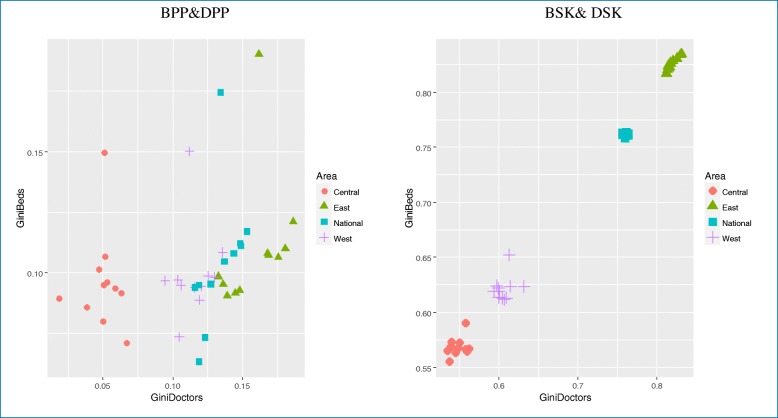
Correlations between Gini coefficients of TCM hospital beds and doctors in different regions. BPP, TCM hospital beds per 10,000 population; BSK, TCM hospital beds per square kilometer; DPP, TCM doctors per 10,000 population; DSK, TCM doctors per square kilometer

Figure 5 shows that correlations between the Gini coefficients of TCM hospital beds per 10,000 population and doctors per 10,000 population were not significant in any regions (all *P* > 0.05). Similarly, correlations in Gini coefficients between TCM hospital beds per square kilometer and doctors per square kilometer were not significant in any regions (*P* > 0.05) except the East (*r* = 0.959, *P* < 0.05).

## Discussion

### Summary of principal findings

Our study observed that all indicators of TCM hospital beds and doctor density have increased over the past 11 years. The number of TCM hospital beds per 10,000 populations increased the fastest. Geographical clustering was not obvious in the density distribution of TCM hospital beds or doctors, as no significant spatial autocorrelation was found. Gini coefficients showed that from 2004 to 2014 the distribution of TCM hospital beds per 10,000 population and doctors per 10,000 populations were equitable between different regions. A large gap existed in the distribution inequality of TCM hospital beds (doctors) per square kilometer among different regions.

### Implications for policy and practice

Our study has several major findings and merits. Firstly, all indicators of TCM hospital beds and doctor density have increased over the past 11 years. The number of TCM hospital beds per 10,000 population grew the fastest. As important indicators of healthcare resources, the density of TCM hospital beds and doctors can provide insight on supply-side investment in health systems, demand-side access to healthcare services, and can ultimately affect inequality in health outcomes. To meet the people’s demand for healthcare, China plans to expand the supply of TCM services, improve TCM health management and ensure that the people can enjoy safe, efficient, and convenient TCM services [28].Our figures also confirm the Chinese government’s effort and input in TCM services. From the outline of strategic planning for the development of TCM [8], the number of beds per 1000 population in public TCM hospitals should reach 0.55, and doctors per 1000 population should reach 0.4 by 2020.After comparing the data in our figures and the goals of the plan, we believe that it will be possible to reach the target number of TCM hospital beds, but difficult to reach the target number of doctors in the remaining 5 years.

Secondly, though the Chinese government emphasizes TCM development and reform, there is still geographic inequity in the distribution of TCM hospital beds and doctors. Gini coefficients showed that from 2004 to 2014, the distribution of TCM hospital beds per 10,000 population and doctors per 10,000 population were equitable across different regions (the East, West, Central and National). A large gap existed in the distribution inequality of TCM hospital beds (doctors) per square kilometer across different regions. Provinces in the East were the most unsatisfactory due to they having the highest Gini coefficient. These findings are similar with those of previous studies which have focused on health resource allocation in China [11, 29]. The absolute number of TCM resources is insufficient to fulfill universal accessibility of health services, and the elevated provision of human resources does not necessarily indicate a decline in inequity, as has been proven in other countries [30]. Despite the large land area and sparse population in Western China, it was in the East where serious inequalities in TCM hospital beds per square kilometer and doctors per square kilometer were found. As enormous differences exist in economic development between the rural and urban areas in the East, it is to be expected that rich health resources are concentrated in the cities, and not the countryside [31]. This phenomenon is more obvious in the East which has experienced the most rapid economic development [32].

Thirdly, geographical clustering was not obvious in either the density distribution of TCM hospital beds or doctors as no significant spatial autocorrelation was found. The geographical clustering means that the “Matthew Effect (accumulated advantage)” in healthcare resource allocation exists. In short, it is convenient for residents of provinces with a high density of TCM hospital beds or doctors to access health services in their own province, as well as in nearby provinces with plentiful healthcare resources. We also observed that correlations in the Gini coefficients between TCM hospital beds and doctors were not significant. This result illustrates that the government has probably made a decision on health policy without considering the cumulative effects of the allocation of the health workforce and the allocation of hospital beds. As the management of TCM hospital beds requires a corresponding health workforce, the equitable allocation of healthcare resources not only includes the absolute number and distribution of resources, but also the balance and coordination of related resources.

### Strengths and limitations of the research

Our study used Chinese provincial-level data from 2004 to 2014 to explore the geographical distribution of TCM hospital beds and doctors, and to explore the inequality in their allocation across different regions. As far as we know, this is the first health equity study of TCM health resource allocation to employ longitudinal data to reveal the trends in temporal and spatial variation of health resource allocation.

However, several limitations in our study should be born in mind. Firstly, this study employed provincial-level data on TCM hospital beds (doctors), not county-level data. The county-level data is more precise and concrete for providing in-depth information and drawing targeted health policy recommendations. As we could not have obtained the county-level data in any way, we think that the provincial-level data is less precise compared to the county-level data. However, we made comparatively deep analyses of provincial-level data, and also arrived at several useful policy implications. Secondly, due to a lack of national survey data, it was impossible for us to separately analyze rural and urban area data in order to control for the area impact. Thirdly, as details such as doctor qualifications, hospital levels and other indicators of TCM service quality could not be found in published reports, our study did not analyze the quality of TCM services. We will complement these in future research. Fourthly, The literature on the culture and values of residents in health equity study of TCM health resource allocation does not exist. We couldn’t make some discussions about this. We believe this is an important area for future research.

## Conclusion

This study has shown that in China all indicators for TCM hospital beds and doctor density have increased over the past 11 years. These results have been reached by employing provincial-level data from 31 provinces. Geographical clustering was not obvious as no significant spatial autocorrelation was found. Great gap existed in the distribution inequality of TCM hospital beds (doctors) per square kilometer among different regions but not for the distribution of TCM hospital beds (doctors) per 10,000 population ratio. Correlations of Gini Coefficients between TCM hospital beds and doctors were not significant. Targeted health policy with equitable distribution of TCM hospital beds (doctors) per square kilometer and the balance and coordination of related resources should be a priority in shaping China’s healthcare system reform.

## Abbreviations

BPP: Beds per 10,000 population
BSK: Beds per square kilometer
DPP: Doctors per 10,000 population
DSK: Doctors per square kilometer
TCM: Traditional Chinese Medicine
